# Communication as a non‐technical skill in the operating room: A qualitative study

**DOI:** 10.1002/nop2.830

**Published:** 2021-02-25

**Authors:** Sisilie Havnås Skråmm, Inger Lise Smith jacobsen, Ingrid Hanssen

**Affiliations:** ^1^ Akershus university hospital Lorenskog Norway; ^2^ Lovisenberg Diaconal University College Oslo Norway

**Keywords:** communication, Non‐technical skills, operating room nursing, patient safety, teamwork

## Abstract

**Aim:**

The aim of this study was to explore how operating room nurses (ORNs) experience operating room (OR) team communication concerning non‐technical skills.

**Design:**

Based on the Scrub Practitioners List of Intraoperative Non‐Technical Skill (SPLINTS), qualitative individual in‐depth semi‐structured interviews were conducted with 11 ORNs in a Norwegian university hospital. Braun and Clarke's six analytic phases for thematic data analysis were used.

**Results:**

Surgeons being unprepared or demanding different instruments than the preoperative information indicates, cause stress and frustration. So does noise and brusquely or poor communication. Ensuring good information flow within the entire team is important. When silence is required, the ORNs communicate with gestures, looks and nods. Creating a positive and secure team culture facilitates discussions, questions and information sharing.

**Conclusion:**

Inappropriate dynamics, inaccurate and/or disrespectful communication and noise may reduce patient safety. Interdisciplinary team training may bring attention to the value of communication as a non‐technical skill.

## 1 INTRODUCTION

In the operating room (OR), communication and teamwork may be complicated skills as the members of the OR teams vary according to the type of surgical procedure. To complicate matters, nurses’ and surgeons’ understanding of the quality of team communication may differ. According to Mills et al. (2008), surgeons tend to describe communication and teamwork as better than nurses and anaesthesiologists do. Surgeons tend to emphasize instrumental communication, that is, the giving and receiving of sufficient information for them to perform the surgical procedure. Instrumental communication is equally important to operating room nurses (ORNs). However, the ORNs are also concerned with interpersonal factors and the importance of the relationship between all health professionals present in the OR to ensure that they function as a well‐functioning team (Leonardsen, 2015).

Information is critical to ensure that the teamwork runs smoothly, without which the surgery cannot be performed efficiently and securely for the patient. Hence, the ORNs’ work is an independent one whether the tasks in hand are technical, non‐technical or collaborative as they in close collaboration with other professionals in the surgical team are responsible for safeguarding the patient.

## 2 BACKGROUND

The background for this study was the introduction of the Scrub Practitioners List of Intraoperative Non‐technical Skills (SPLINTS) (Mitchell & Flin, 2008a) in a Norwegian university hospital. The purpose of the SPLINTS project was to identify the non‐technical skills necessary for safe and effective work performance by the ORNs through focusing on the SPLINTS assessment tool's three main taxonomical categories: 1 Situational awareness (perception of environmental elements and comprehension of their meaning), 2. Communication and teamwork (acting assertively; communicate information; coordinating with others), and 3. Decision‐making and leadership (selecting a required option to deal with the situation) (Vogelsang et al., 2020).

Mitchell and Flin (2008a) describe non‐technical skills as cognitive and social skills that are complementary to technical skills. Cognitive skills refer to how you think in routine and emergency situations including decision‐making and situation awareness. Pires et al. (2017) hold that non‐technical skills are considered particularly important to prevent errors. Regardless of the complexity of the procedure, non‐technical skills are fundamental to good teamwork and thus essential to patient safety (Flin et al., 2007; Høyland et al., 2011; Youngson & Flin, 2010).

Shared information and knowledge within the OR team are the basis for obtaining the common understanding of purpose and goals needed to secure progress throughout the surgical procedure (Mitchell & Flin, 2008b). Furthermore, precise and constructive communication builds respect, trust, recognition and acceptance within the team (Kaldheim & Slettebø, 2016; Lauvås & Lauvås, 2004). Successful surgical procedures depend on shared information between physicians and nurses, and that both parties integrate knowledge and skills in their communication and collaboration (Healey et al., 2006). Thus, communication as a non‐technical skill complements technical skills in handling tasks in an efficient and secure manner. Through communication trusting interpersonal relations and good organization are developed, factors which together with education are important for successful interdisciplinary teamwork (Gillespie et al., 2010).

The aim of this study was to explore how ORNs experience OR team communication concerning non‐technical skills. The research question related to this paper was as follows: How do operating room nurses experience the non‐technical skills communication and teamwork in the operating room to ensure patient safety?

## 3 DESIGN AND METHOD

This study has a qualitative design where individual in‐depth interviews were conducted to learn how ORNs in a central perioperative unit in a Norwegian university hospital experienced the use of communication concerning non‐technical skills in the OR team in light of the SPLINTS assessment tool (Flin et al., 2014). The central perioperative unit in question has about 90 perioperative nurses, 14 operating rooms and seven sub‐units or specialties: gynaecology, vascular/thorax, gastroenterology, urology, ear/nose/throat, endocrinology and orthopaedics.

A qualitative semi‐structured interview guide was used based on SPLINTS’ three main areas. In this paper, SPLINTS’ main area 2, communication and teamwork, is focused. This pertains to acting assertively, exchanging information and coordinating with others.

### 3.1 Data collection

Before the interviews took place, all the unit's ORNs were informed about the study. Of the 47 nurses who fulfilled the inclusion criteria, 11 accepted the invitation to participate in the study. Their names were given to the first and second authors through their respective sub‐unit heads. The interviews were conducted in a quiet room in the hospital's perioperative unit with only the interviewer and the interviewee present. All the interviews were performed during the respective interviewee's day shifts.

*Inclusion criteria:* ORNs with more than five years experience as supervisors for perioperative nursing students. There were no specific exclusion criteria.

As seen in Table 1, the mean age of our interviewees was 52 years.

**TABLE 1:** Overview of the studies’ participants

Interviewees	Age	Average no. of years as registered nurses	Average no. of years as perioperative nurses
4	40–50 years	18 years	12 years
5	51–60 years	21 years	12 years
2	60 + years	36,5 years	27,5 years

The mean age of Norwegian ORNs was 51.8 years in 2013 (Dolonen, 2013).

What the concept “non‐technical skills” entails was discussed with the interviewees before the interviews were commenced. The interviews, lasting 21–79 min, took form of an electronically recorded talk where the interviewees were encouraged to recount their experiences.

### 3.2 Interview guide

Regarding communication and teamwork, the interviewees were asked the following questions:

- How do you cooperate and exchange information within the team/with co‐workers?
- How do you experience the communication with other team members in various situations?
- Describe factors that may influence the communication within the OR team.

### 3.3 ETHICAL CONSIDERATIONS

The project was approved by the Norwegian Centre for Research Data in care of the hospital's Data Protection Officer. The interviewees were informed in writing and orally that participation was voluntary and that they were free to withdraw from the project whenever they wanted without giving any explanation. They all gave their written informed consent to participate. Interview transcriptions are stored safely according to Ethical Research Guidelines (Helsedirektoratet, 2009). Recorded interviews were deleted after transcription.

### 3.4 Data analysis

The first author conducted the interviews. She and the second author transcribed the interviews verbatim. These two authors are both ORNs, while the third author is not and therefore had an outsider view on the data. Thus, we tried to minimize bias and strengthen trustworthiness. All three authors took part in the data analysis which was thematic and hermeneutic in character where depth of understanding was attained through a circular investigation of the interviews (Gadamer, 2012). Braun and Clarke’s (2006) six analytic phases for thematic analysis were used: 1) The authors familiarized themselves with the data. 2) Interesting features were coded and collated into potential themes (phase 3, searching for themes). Phases 4 (reviewing themes) and 5 (defining and naming themes) (Braun & Clarke, 2006) were done collaboratively by all the authors. 6) The first author wrote a preliminary paper text which then was discussed and developed further collaboratively. All the while, we tried to be open, curious, communicate authentically, and to realize that the fusion of horizons through the reading of texts leads to the creation of something new (Gadamer, 2012) and to avoid bias. Both the first and second authors have previous qualitative researcher experience. Even so, a professor of nursing, well versed in qualitative research, was invited in as co‐analyst, co‐author and mentor. This was done to avoid analytic bias and to add depth of reflection to the analyses.

### 3.5 Trustworthiness

Trustworthiness and rigour were obtained through following Braun and Clarke’s (2006) phases of thematic analysis while we read and re‐read the interview texts and thus strived to “remain open to the meaning of the other person or the text” (Gadamer, 1989, p. 268).

The study's credibility is ensured through the choice of context, participants and research approach suitable for the focus of our study. Quotations/telling meaning units are presented to emphasize our findings (Polit & Beck, 2014). This also strengthens the study's confirmability as it shows that the findings are based on our interviewees’ responses and not on potential bias or any personal motivations that would skew our interpretations (ibid). Dependability is achieved through interviewing ORNs with varied and extensive experience, presenting the basic questions asked during the interviews, and following the chosen model for data analysis step by step. Thus, it will be possible to repeat the study in a similar OR setting by other researchers and acquire findings in line with ours. And finally, trustworthiness through transferability is achieved by presenting thick descriptions to show that the study findings can be applicable to other OR similar contexts, circumstances and situations.

## 4 RESULTS

Central in the interviews are factors that may influence communication and the exchange of information within the OR team.

### 4.1 Factors that may influence communication

According to several of the interviewees, some surgeons take it upon themselves to define who may speak in the OR and who may not. Others restrict their communication to barking commands. Sometimes surgeons communicate important information too late for the ORN to have the correct instruments—or rather—the surgeons’ *preferred* instruments available. Or they may have a brusque way of imparting information and orders, something which may create uncertainty within the team. Nurse #7 even finds that she has to “be prepared to ‘accept’ unpleasant communication to maintain a good atmosphere in the OR.”

This kind of unidirectional and hierarchical communication influences the OR team in a negative way. Experienced ORNs have learned to handle these surgeons, but found them rather daunting when they were new to this field of nursing. As Nurse #1 put it: “Experienced operating room nurses find it easier to be heard than those who are new. With experience it is easier to speak your mind.” This latter point is important as the ORNs regard themselves as the patients’ advocate. It is essential for them to argue for the patients’ needs with the surgeons or the anaesthetists when necessary, for example during long‐lasting surgeries where change of the patient's positioning is needed to prevent complications.

The interviewees emphasize the potential negative effect on communication and teamwork if team members lack understanding of each other's responsibilities: “To know the team members and to know how the surgeon wants things done make communication and the practical workflow easier” (#3). Insight into the preferred routines of each of the surgeons improves communication and reduces stress. Surgeons who are not properly prepared or suddenly want different instruments than the preoperative information indicates, create stress, cost valuable time and cause frustration and negative communication: “If some people are stressed it influences the communication [very much]” (**#**11).

Several surgeons working together may enhance this challenge as it impairs the situational overview the ORNs need to be effective and efficient assistants. At times some surgeons have an internal conversation going and then they suddenly may give orders without changing the volume or pitch of voice, making the order difficult to catch. The interviewees saw “mumblers” and foreign co‐workers who master the Norwegian language poorly as particularly problematic: “And of course, if there are some with a foreign language that makes it hard to communicate, this may create difficulties.” (#11).

Noise also impairs team communication. Several interviewees mention this in connection with the completion of large and complicated surgical procedures, particularly when “we are to count equipment and instruments.” Then the noise level tends to be very high with “a lot of unnecessary unrelated talk. And we are two operating room nurses who are to count these things but are not given the quiet and the time to do the job. And it is a job we have to finish before the patient is taken to the post‐operative unit” (#4).

### 4.2 Exchange of information within the OR team

Securing correct preoperative information about the patient and the surgical procedure is pointed out as essential for being prepared for the work that is to be done in the OR. Insufficient preparation may influence the result negatively. The notes written by the surgeon the previous day are therefore considered as very important. When “the scheduled surgery has a different surgeon than planned and the procedure turns out to be different from the information we are given” (#3), this interferes with the ORNs’ work as both the positioning and the surgical draping of the patient might have to be changed after the arrival of the surgeon. When in doubt, the ORNs often call the surgeon beforehand to make sure that everything is according to his or her preferences.

The patient is him‐/herself an important source of information. Except for those who are being operated in regional and local anaesthesia, most patients are awake only for a limited time in the OR ahead of surgery. In this often brief period, the interviewees try to learn as much as possible from the patients that may be important for the procedure: “I ask the patients if they for instance have a total hip or knee replacement and other issues regarding the body related to how they have to be positioned on the table. I use the time while moving the patient from the preoperative room into the OR to talk about such matters. This way a conversation is started, and the patient often spontaneously tells me things that help me understand what is important to this person in this particular situation” (#4).

To mediate information between the members of the OR team perioperatively is perceived as equally important. This includes “all the various interdisciplinary discussions. It is very important to share this with the entire team so that everyone is informed and understands that we were going in that direction, but now we have turned forty‐five degrees to the right because we now have decided to do something different because of this and that. So that everyone is up to speed” (#4).

During particularly complicated or precarious operations, the ORNs do their best not to disturb the surgeons by asking questions. When oral communication is difficult, for instance because of noise, or talk for some reason should be avoided “I look at my colleague who is assisting me: Did you understand that we need different equipment? And she merely nods. When we are two who have worked together for years, it is a very good feeling. Most things are done by looks and small gestures … discrete hand movements without the use of much energy. We look at each other and agree on things. I find that to be a good way of communicating” (#4).

A friendly atmosphere opens up for the asking of questions. In such an atmosphere the interviewees find it easier to share information and even point out errors made by team members. It furthermore makes the ORNs feel appreciated and creates a positive team feeling.

## 5 DISCUSSION

The operating room is a highly technical work environment where the attention needs to be on patient care and safety as well as on surgical or other invasive procedures. This current study's most important finding is that communication in the OR has considerable impact on the OR team's performance and through this on patient safety and treatment outcome. The outcome of a given surgery is influenced by the collaboration within the surgical team, each team member's competency, and the tasks in hand (Schmutz et al., 2019). Sexton et al. (2018) hold that perioperative and post‐operative injuries are generally caused by human factors and human errors. The surgical team's non‐technical skills are associated with patient safety through efficient collaboration and trust within the team. The workplace culture may vary from one hospital to the next which may affect the way non‐technical skills are discussed and handled.

Healthcare settings are hierarchical environments. Our interviewees indicated that some surgeons could be condescending and even disrespectful. This is also described by Tørring et al. (2019) who during observations of perioperative teams found that “[s]ometimes communication between team members was inappropriate, and sometimes the tone of voice was ambiguous and disrespectful.”

Tørring et al. (2019) point to the importance of “mutual respect, supported by frequent, timely, accurate, and problem‐solving rather than blaming communication.” They claim that this leads to “higher levels of quality, efficiency, and job satisfaction as well as work engagement, psychological safety and the ability to learn from errors.” The importance of respectful, adequate and informative communication is supported by Penprase, Elstun Ferguson, Schaper and Tiller (2010) who connect it to patient safety, reduction of uncertainty within the perioperative team and the promotion of harmonious teamwork which enhances efficient care and job satisfaction. This is in line with our findings.

While good communication reduces stress, uncertainty inhibits communication and causes stress. Leonard, Graham and Boacum (2004) hold that “[h]ierarchy, or power distance, frequently inhibits people from speaking up.” Their study shows that condescending and disrespectful communication may be the result of a power distance that tends to create lack of psychological safety, unhealthy cultural norms and uncertainty as to the plan of action. Furthermore, tension among team members that negatively influence communication and collaboration is among the factors that may lead to procedural errors (Garrett, 2016; Jenkins, 2015; Lingard et al., 2004; Penprase et al., 2010; Pires et al., 2017) and patient harm (Høyland et al., 2011; Youngson & Flin, 2010).

Our interviewees emphasized the importance of the surgical team members understanding each other's responsibilities. They also found it useful to know how individual surgeons wanted the OR prepared for various specialized surgeries as this created a positive “flow.” Also Tørring et al.’s (2019) ORN participants found it helpful to know “one another's role and expertise and took into account what was important for each other's task execution.” In line with this, Gillespie et al. (2012) study shows that lack of knowledge about one another within the perioperative team increased the likelihood of miscommunication and interruption during surgical procedures. Surgical teams, at least in Norway, tend to be established ad hoc, with different team members from day to day. Furthermore, there tend to be frequent changes in the surgical schedules. Both these factors may impair the quality and effectiveness of performance. Good teamwork in interdisciplinary surgical teams is challenged by interdependence, time constraints and uncertainty (Torring et al., 2019) as unexpected situations may occur.

To avoid miscommunication and interruptions, the ORNs need continually to divide their focus of attention between the execution of their individual assignments and the coordination of the team. Thus, all perioperative team members are enabled to effectively manage their roles and responsibilities (Garrett, 2016). The sharing of knowledge within the team is required, something which is challenged by time constraints and if team members feel insecure in the surgical context (Leonard et al., 2004; Torring et al., 2019). Lingard et al. (2004) found that communication failures caused by contextual problems were common and generally entailed an exchange of information at the wrong time—typically, too late to be of any use. This resembles the experiences described by our interviewees when the surgeons keep an internal conversation going and then suddenly give orders without changing the volume or pitch of voice. Because of this, the ORN may miss that the last thing said was meant for her ears. Such unclear communication may cause problems as it is difficult to catch.

Surgeons tend to report higher satisfaction with the teamwork climate and communication than nurses, and they experience communication and teamwork different from the rest of the OR team (Mills et al., 2008; Sexton et al., 2000). They also tend to describe a stronger safety culture than other team members. The reason behind this may be a gap in communication styles between nurses and physicians (Sexton et al., 2000).

Differences in communication styles are not the only problem, however. Many of our interviewees held that noise impaired team communication during surgery. Several also mentioned noise in connection with the counting of instruments at the completion of surgical procedures. This is perceived as a stressor and something that potentially may influence patient safety negatively. In the interest of patient safety, it is important for the ORNs to be able to concentrate on their work despite the immense demands on their attention (Ingvarsdottir & Halldorsdottir, 2018).

Tørring et al. (2019) found that surgical teams that communicated proactively and collaborated dynamically were characterized by a broad consensus concerning shared goals, a noticeable expression of mutual respect, and timely and accurate communication focused on solving the problems at hand. Together, these team members searched for the best possible solutions and made appropriate decisions. Depending on the surgery and team dynamics, communication during many operations may be characterized by verbal silence. According to Tørring et al., (2019), this type of silent interpersonal dynamic appeared when the team members performed safe‐surgery procedures. As in our study, the verbal exchange of information during these procedures was often very brief.

Our interviewees worried that insufficient preparation could influence negatively on the outcome of the surgical procedure. They found that unexpected changes of surgeons or procedures created stress and last‐minute reorganization and replacement of instruments to suit the new setting. If they became aware of these changes in time, they would call the surgeon to receive the information they needed. Otherwise, they had to do the changes after the surgeon was ready to start. These were problems also seen by Tørring et al. (2019) during their observations of ORNs. Last‐minute changes could leave the ORNs unprepared to follow the surgeons’ moves during the surgical procedure, or they could have problems getting hold of the surgeons prior to surgery. This resulted in prolongation of ongoing surgery or in delays.

Tørring et al. (2019) point to the need of inter‐team training programmes to improve communication and interdisciplinary collaboration in the OR. Also, evaluation of the communication in the OR can be helpful to improve team collaboration. Here, the assessment tool SPLINTS may be helpful. SPLINTS enables retrospective analysis of the surgical team's communication and what factors influenced the communication (Flin et al., ,,,,,,,,^2008^, ^2014^). Such an analytic approach to “real‐life” contexts together with interdisciplinary team training may bring attention to the value of communication as a non‐technical skill and how this affects team performance, patient safety and treatment outcomes (Ballangrud et al., 2014).

## 6 CONCLUSION

Based on our findings, we will hold that the outcome of a given surgery is influenced by the collaboration and communication within the surgical team. We find that communication and teamwork are closely associated with patient safety. Inappropriate dynamics, inaccurate and disrespectful communication patterns may disturb effective communication and reduce patient safety. Negative communication styles and noise in the OR may disturb communication and thus the teamwork. Insufficient preparation or unexpected last‐minute changes may furthermore result in prolongation of ongoing surgery or delays, or even influence negatively on the outcome of the surgical procedure. Our study indicates that the assessment tool SPLINTS may be helpful in evaluating the communication in the OR and thus be helpful in improving team collaboration.

### 6.1 Limitations

A clear limitation in this study is that only 23% (11 of 47) of potential interviewees chose to participate in the study. There is furthermore a danger of selection bias as operating room nurses with less than five years’ experience mentoring perioperative students were excluded. This choice was based on the primary aim of the study as a whole. According to Benner (1984), less experienced nurses may focus more on technical skills and on doing things “by the book”, which might have given somewhat different answers.

The fact that this is a single unit study limits the study as workplace cultures may vary, and interviews conducted in other hospitals may have given different results. Although two male interviewees reflect the male proportion of operating room nurses in Norway, it is an inadequate number of interviewees to study possible gender differences in how experiences and feelings are perceived and expressed.

Both the first and the second authors work in the perioperative unit in question, the first author, who conducted the interviews, in a supervisory capacity. This may have influenced the interviewees’ willingness to give frank descriptions of their experiences and thus have affected the interview conversations and the credibility of the study. To what extent the interviewees’ feeling of loyalty and/or dependence on the interviewer as a representative of the unit's middle‐range leadership may have impacted on the results is difficult to say. To minimize bias and strengthen trustworthiness as far as possible, the third author, a nursing professor with no connection to either the unit or the interviewees, was invited into the project as mentor, co‐data analyser and co‐author. Analysis of the interviews seems to indicate that the interviewees have been frank in their responses as they did not seem to hesitate to criticize organizational and leadership issues during the interviews.

## CONFLICT OF INTEREST

All authors declare they have no conflict of interest.

## **AUTHOR** **CONTRIBUTIONS**

SHS, ILSJ and IH: Substantial contributions to conception and design, or acquisition of data, or analysis and interpretation of data; drafting the manuscript or revising it critically for important intellectual content; final approval of the version to be published, and agreeing to be accountable for all aspects of the work in ensuring that questions related to the accuracy or integrity of any part of the work are appropriately investigated and resolved. Each author should have participated sufficiently in the work to take public responsibility for appropriate portions of the content.

## Data Availability

The authors confirm that the data supporting the findings of this study are available within this article. The data were generated at Akershus University Hospital. In accordance with the research approval given by the Norwegian Centre for Research Data in care of the hospital's Data Protection Officer, the raw data may not be shared with persons outside the research group. However, the first author will answer questions regarding the study and its data on a general basis: sskr@ahus.no.
